# Survival and *NF1* Analysis in a Cohort of Orthopedics Patients with Malignant Peripheral Nerve Sheath Tumors

**DOI:** 10.1155/2021/9386823

**Published:** 2021-10-04

**Authors:** Daniel K. Knewitz, Colin J. Anderson, William T. Presley, MaryBeth Horodyski, Mark T. Scarborough, Margaret R. Wallace

**Affiliations:** ^1^University of Florida College of Medicine, MS3, Gainesville, FL, USA; ^2^Department of Orthopaedics and Rehabilitation, University of Florida College of Medicine, Gainesville, FL, USA; ^3^Department of Orthopedic Surgery, Levine Cancer Institute and Musculoskeletal Institute, Carolinas Medical Center–Atrium Health, Charlotte, NC, USA; ^4^Department of Molecular Genetics & Microbiology, University of Florida College of Medicine, Gainesville, FL, USA; ^5^University of Florida Health Cancer Center, Gainesville, FL, USA; ^6^University of Florida Genetics Institute, Gainesville, FL, USA

## Abstract

Neurofibromatosis type 1 (NF1) is an autosomal dominant tumor syndrome in which benign plexiform neurofibromas are at risk of transforming into malignant peripheral nerve sheath tumors (MPNSTs), a very rare soft-tissue sarcoma. The prognosis of patients with MPNSTs is poor, with most studies reporting <50% survival at five years. However, studies evaluating MPNSTs are limited and report heterogeneous results. Because no MPNST-specific evidence-based treatment guideline exists, individual institutional experiences are very informative to the field. The main objective of this study was to investigate and report MPNST prognostic clinical and genetic biomarkers from our institution's Orthopedics service experience treating 20 cases from 1992 to 2017. Most patients were treated with resection and adjuvant radiation. Extended follow-up, averaging 11.4 years (ranging 1.1 to 25.1), revealed excellent five-year survival rates: 70% for overall and 60% for metastatic disease. An S100 B immunonegative tumor phenotype was associated with a significantly worse outcome than MPNSTs with positive S100 B stain. In addition, *NF1* gene mutation analysis was performed on 27 families with NF1 in which at least one affected family member developed MPNSTs. Of the 27 *NF1* germline mutations, five were large deletions spanning (or nearly spanning) the gene (18.5%), substantially more than such deletions in NF1 in general, consistent with increased risk of MPNSTs in such cases.

## 1. Introduction

Neurofibromatosis type 1 (NF1) is an incurable progressive autosomal dominant disease. Despite an NF1 incidence of 1 in 3000 people, the first effective systemic therapy with the MEK inhibitor selumetinib was only recently FDA approved, in 2020 [1]. NF1 is caused by heterozygous germline mutations in the *NF1* gene, of which over 5000 have been identified spanning the large locus. The clinical hallmark of NF1 is the neurofibroma, a benign peripheral nerve sheath Schwann-cell tumor. Patients can develop few to thousands of neurofibromas in their lifetimes. Neurofibromas are classified as “cutaneous” when they are superficial, involving nerve endings in the skin; larger and usually deeper “plexiform neurofibromas” involve peripheral nerves [2]. Plexiform neurofibromas are thought to be congenital in origin and occur in about 50% of patients [2]. Plexiform neurofibromas can grow quite large and are estimated to have a 10–30% risk of malignant transformation into a malignant peripheral nerve sheath tumor—a rare type of soft-tissue sarcoma—particularly if they have a nodular phenotype [2]. MPNSTs are rare, affecting only 1.4 in 100,000 people, with half of such cases occurring in NF1 patients [3]. MPNSTs are not reported to show a predilection toward a specific anatomical region of the body. Cutaneous cases of MPNSTs have been described, but they do not typically occur in NF1 patients [4]. Overall, the lifetime risk of MPNSTs in NF1 patients is estimated at 12–15% [5]. In reported studies, the MPNST 5-year survival rate is usually reported as less than 50% [6] and thus the prognosis is generally considered poor.

Because of its rarity, there have been few clinical trials specifically for patients with MPNSTs and information about natural history and response to therapies in the literature is sparse. Typically, MPNSTs are managed with treatment protocols for soft-tissue sarcomas, as protocols specific to MPNSTs have not been established [7]. Early surgery has been shown to be an effective treatment [8] with a goal of resecting the tumor with wide or negative margins. Adjuvant radiation or chemotherapy regimens have also been used at different institutions to reduce the risk of local recurrence or to treat systemic disease [7]. Although recent trials with novel targeted therapies have so far proven ineffective, recurrent gene mutations that have been associated with more aggressive tumors in patients with NF1, such as the polycomb repressive complex 2 (PRC2) core components, embryonic ectoderm development protein (EED), and suppressor of zeste 12 homolog (SUZ12), may serve as future treatment targets [9–12]. There is some controversy in the literature regarding germline *NF1* mutation effect on the risk of developing an MPNST. Some studies reported a higher incidence of deletions spanning the entire *NF1* locus and surrounding genes in NF1 patients who develop an MPNST than the NF1 population overall [13], while other reports did not find such a trend [14].

There are a limited number of publications reporting MPNST survival, most with small case numbers and variable outcomes, not unexpected for such a rare tumor. Thus, experience from institutions managing patients with MPNSTs is important to add to the field's knowledge [15]. The purpose of our study was to evaluate both clinical and *NF1* mutation data from the MPNST population at our institution to identify prognostic factors.

## 2. Materials and Methods

### 2.1. Clinical and Demographic Data Collection

As approved by the University of Florida Institutional Review Board, a retrospective medical chart review of 1992 to 2017 was performed from 1992 to 2017 to gather data on our institution's Division of Orthopedic Oncology's experience of treating patients with MPNSTs. The data were extracted from an EPIC electronic medical record service and from the Enneking/Anspach Research Center database in the Department of Orthopedics and Rehabilitation. Inclusion criteria were (1) diagnosis of MPNSTs, (2) definitive treatment performed at our institution, and (3) at least 12 months of follow-up data. Demographic and clinical information collection included gender, self-reported ethnic group, age at initial diagnosis, tumor stage at first diagnosis, time to recurrence, diagnosis of NF1, family history of NF1, therapeutic history, immunohistochemical S100 B data from a pathology service, and survival status.

### 2.2. *NF1* Gene Mutation Analysis

*NF1* mutation analysis was performed on DNA samples from the IRB-approved Wallace Genetics Bank from 27 NF1 families bearing at least one MPNST occurrence. Previously existing data included some *NF1* cDNA Sanger sequencing, while current testing included PCR and sequencing of 13 exons (4, 5, 11, 13, 14, 16, 18, 20, 22, 26, 28, 32, 40, and 46) of the 57 exons in the locus (NCBI gene sequence: NG_009018.1) (methods described in [16, 17]). In addition, all samples were screened for large deletions using four TaqMan *NF1* copy-number assays across the gene (ThermoFisher; Hs05477010, Hs06413401, Hs05512625, and Hs03960106) (Center for Pharmacogenomics Core, UF Clinical and Translational Institute).

### 2.3. Statistical Analysis

All data were analyzed using statistical software (SPSS 25, IBM Co. Armonk, NY). The significance level for analyses was set *a priori* at *p* < 0.05. Descriptive statistics were computed for demographic and clinical variables. Kaplan–Meier survival curves were generated to assess the overall survival of the study population and compare between demographic, clinical, survival, and pathological variables.

## 3. Results

A total of twenty subjects (14 males and 6 females) qualified for this study's retrospective chart review. Demographic characteristics are provided in Table 1.

**Figure 1:**
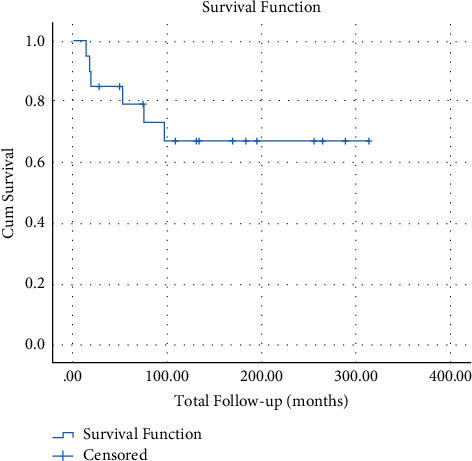
Kaplan–Meier survival curve for all 20 subjects. The 5-year survival was approximately 70%.

With respect to race, 12, 6, and 1 subject(s) were Caucasian, black, and Hispanic, respectively. Race was not identified in the medical record of one subject. The mean total length of follow-up was 136.9 months. The survival rate for the entire study population was approximately 70% (Figure 1).

Two subjects had local recurrence, and five had subsequent metastasis. The mean time to local recurrence and time to metastasis were 134.9 and 113.6 months, respectively. Both subjects with local recurrence received radiation therapy. At the last follow-up for all subjects, 14 were alive and 6 were deceased. Two of the five subjects who developed subsequent metastasis were alive at the last follow-up (2018) and survived an average of 197.9 months (range 85.7 to 309.8) after diagnosis of metastasis. Four of these metastases targeted the lung, and one subject developed metastasis in the brain. The five-year survival rate of patients with metastatic disease was 60%.

The female gender was associated with better cumulative survival outcomes, and the difference was not significant (*p*=0.45). Black subjects were found to have better cumulative survival outcomes than Caucasian and Hispanic subjects; however, the difference was not significant (*p*=0.25). The most common primary MPNST locations in this patient population were the lower extremities (*n* = 11) and the upper extremities (*n* = 8), followed by the neck (*n* = 1). Six subjects had a diagnosis of NF1. Five subjects were NF1-negative, and the NF1 status for nine subjects was indeterminate. Although subjects with negative NF1 status were found to have better cumulative survival outcomes, the difference was not significant (*p*=0.39).

**Figure 2:**
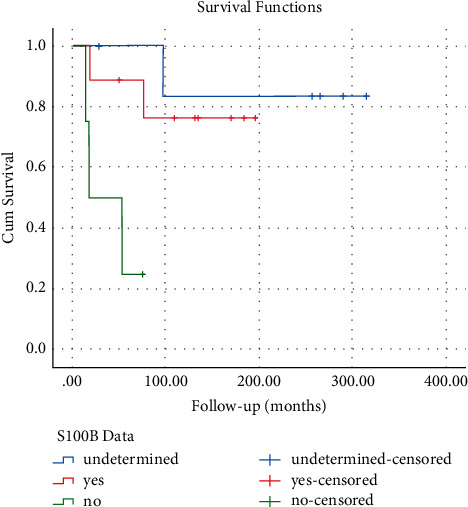
Kaplan–Meier survival curve based on MPNST S100 B status. S100B-negative MPNSTs (green line, *n* = 4) were associated with a significantly lower survival than S100B-positive tumors (red line, *n* = 9) (*p* < 0.003).

Tumor-grade classifications were low (*n* = 2), intermediate (*n* = 3), and high (*n* = 15). Low-grade tumors were found to have better cumulative survival than intermediate and high, although the difference was not significant (*p*=0.63). AJCC 8th Education Stage classification of the patients was as follows: IA = 2, II = 4, IIIA = 8, IIIB = 6. Patients with stage IA and II tumors were all alive at the last follow-up. Overall comparison between stages failed to reveal a significant difference in cumulative survival (*p*=0.26). S100 B immunohistochemistry status was reported positive in 9 tumors, negative in 4, and could not be determined in 7. Subjects with positive S100 B status were shown to have a significantly better cumulative survival outcome (*p*=0.003) (Figure 2).

Three subjects were treated with resection alone (1 subsequently developed metastasis), 15 were treated with resection and adjuvant radiation, and two subjects were treated with resection and chemotherapy. Only one of the resections involved an amputation. When comparing cumulative survival in subjects who received chemotherapy (*n* = 2) against subjects who did not receive chemotherapy (*n* = 18), the two subjects receiving chemotherapy had a poorer outcome, although it was nonsignificant (*p*=0.69). When comparing estimated overall survival in subjects who received radiation (234.1 months) against those who did not (193.9 months), subjects who received radiation had better survival, but the difference was not significant (*p*=0.89).

Among banked DNAs, there were 27 NF1 cases who developed MPNSTs and/or had a positive family history of MPNSTs. As shown in Table 2, germline *NF1* mutations among these 27 families were distributed as follows: nonsense (9), missense (4), frameshift (5), splicing (5), deletion spanning most of the gene (1), and whole-gene deletion (4).

## 4. Discussion

The purpose of this study was to evaluate clinical prognostic factors and genomic biomarkers in patients with MPNSTs treated at our institution. As an academic tertiary-care institution, our institution is a referral center for cancer patients, including those with MPNSTs, from throughout the US as well as internationally. Most similar studies have reported less than a 50% five-year MPNST survival rate [18–30]. Our cohort had a mean follow-up time of 11.4 years and a 70% five-year survival rate, the latter of which is superior to any previously reported studies (range 38.3–62.5 years) [18, 19, 22–30].

Consistent with previous reports, we noted that subjects with S100B-positive tumors had significantly better outcomes than those with S100B-negative tumors [12]. S100 B is a mature Schwann-cell protein, and loss of immunostaining is consistent with poorer cell differentiation. Further investigation is needed to evaluate the use of this phenotype to stratify patient risk, along with immunostaining of other markers in MPNSTs that are of recent interest such as polycomb repressor complex 2 epigenetic mark H3K27me3 [31] and HMGA2 [32].

The frequency of whole-gene deletions (14.8%) in the 27 germline *NF1* mutations associated with MPNSTs was higher in our population than the 4–5% rate of such deletions in NF1 patients in general [33]. This frequency is even greater when including samples with a deletion of most of the gene, extending beyond the 3′ end for an unknown distance. Our result is consistent with previous reports suggesting that individuals with such deletions are at a greater risk for MPNSTs [13, 34, 35]. However, this elevated risk could be related to the increased plexiform neurofibroma burden of many such patients [33].

Although a significant difference in survival was not found based on presence of an NF1 diagnosis, patients with NF1 trended toward a poorer outcome, particularly males: one was still alive at the last follow-up at 314.8 months after metastasis, but three (including one with metastatic disease) had survival under five years (52.43 months after metastasis, 13.74 months after diagnosis, and 17.23 months after diagnosis, respectively). The two NF1 female subjects are both still alive (108.78 months after metastasis and 256.61 months after diagnosis). This study was underpowered to detect relationships between race, gender, and other factors with respect to survival. Similar studies with larger cohorts found individual variables associated with survival, such as tumor size [18, 19, 22, 29], margin status [18, 24–28], NF1 diagnostic status [19, 22, 26, 29, 30], and tumor grade [19, 21, 22, 28].

Our study had a number of limitations, the foremost being sample size and another being lack of NF1 status in earlier cases. Although a larger sample size might aid in identifying additional significant prognostic factors, it is important to report individual institution experiences because of the lack of evidence-based established therapeutic regimens for MPNST. Information from institutions with better outcomes may be very useful to other groups. Toward this goal, we excluded patients who had received prior MPNST treatment at outside institutions. Although this allowed evaluation of survival outcomes based solely on care at our institution, a more varied subject group might have been more representative of the overall US MPNST patient population experience.

## 5. Conclusions

Given the rarity and lack of clinical trial data for MPNSTs, treatment parameters and survival data from multiple institutions are needed to move toward improved treatment and prognosis. It is critical that patients with rare tumors such as MPNSTs be referred to institutions experienced with such cancers. Our institutional experience with MPNST cases treated in the Orthopedics oncology service, with treatment favoring surgical resection and adjuvant radiation, showed an outstanding five-year survival of 70%. This work also highlighted the potential use of S100 B immunostaining in prognosis. In addition, our study added to the evidence that whole-gene germline *NF1* deletions are associated with an increased risk of MPNST.

## Figures and Tables

**Table 1:** Subject demographics and clinical survival information.

	Mean	Std. dev.	Range	Minimum	Maximum
Age at diagnosis (years)	43.4	18.4	75.4	6.0	81.4
Time to local recurrence or last follow-up (months)	134.9	102.1	309.9	4.9	314.8
Time to metastasis or last follow-up (months)	113.6	100.0	284.8	4.9	289.7
Total follow-up (months)	136.9	99.8	301.1	13.7	314.8

**Table 2:** MPNST-related *NF1* germline mutations.

Mutation	Effect	Exon
c.499delTGTT	Frameshift	5
c.958 G > Cp.Ala320Pro	Missense	9
c.1039 C > Tp.Gln347X	Nonsense	9
c.1782delAG	Frameshift	16
c.2325 G > *A*	Splice error	19
c.2352 G > Ap.Trp784X	Nonsense	20
c.2446 C > Tp.Arg816X	Nonsense	21
c.2534insG	Frameshift	21
c.2540 T > Cp.Leu847Pro	Missense	21
c.2991-1G > *A*	Splice error	23
c.3113+1G > *C*	Splice error	23
c.3456_3457insA	Frameshift	26
C. 3683delC	Frameshift	27
c.3826 C > Tp.Arg1276X	Nonsense	28
c.4255 A > Tp.Lys1419X	Nonsense	32
c.4435 A > *G*	Splice error	34
c.4868 A > Tp.Asn1623Val	Missense	37
c.5242 C > Tp.Arg1748X	Nonsense	38
c.5914 C > Tp.Gln1981X	Nonsense	40
c.6148 C > Tp.Gln2050X	Nonsense	42
c.6302 C > Gp.Thr2101Arg	Missense	42
c.7285 C > Tp.Arg2429X	Nonsense	50
Whole-gene deletion (*n* = 4)	Null	All
Large intragenic gene deletion (*n* = 1)	Likely null	2–50

*n* = 27. NCBI RefSeq: NM_0000267.

## Data Availability

This study utilized data derived from patient records at our institution; hence, the data are not available for public release. All pertinent HIPAA-compliant data are published in the accompanying tables and figures.
